# Implantation

**Published:** 1887-01

**Authors:** 


### IMPLANTATION.

In the number for November, 1886, we gave an account of a case of implantation performed in this city by Dr. W. J. Younger. Our readers will, perhaps, remember that the operation was in some respects extraordinary, the implanted tooth having been selected from a receptacle for extracted teeth, where it had lain for some years, the exact date of its removal not being known. It has been under observation since its insertion, and we now have to report that it still remains in a comfortable condition, and that no symptoms of irritation or inflammation have manifested themselves. It has not, however, become as firm as the other teeth, and, so far as we can observe, there has been no osseous deposition about it. It was not ligated nor held in position by any external appliance, but simply left to chance and the tender mercies of mother nature, and it remains about as it was when inserted. Our impression is that the tooth is tolerated, rather than adopted into the dental family. We have not prejudiced the chances for final success by rude attempts to see whether anything like a firm connection has been established, and therefore cannot positively determine its exact condition. It is apparently quite as firm as is a tooth in a rather advanced state of pericemental inflammation, but there is no soreness or pain about it.

We are impressed with the conviction that, while the operation for its implantation was skillfully performed, the tooth was not given a fair chance. To simply make a hole and insert a foreign tooth is not a scientific operation, unless all the means to secure the desired result are exhausted. To submit such a case to all the violence which is inseparable from every day use, and to place about it no safeguards against accident, seem to us an invitation to defeat. A freshly implanted tree cannot grow if it be subjected to constant movements of its roots, nor can nature produce new granulations about an organ that is not allowed rest. This is an axiom in pathological science. A transplanted or a replanted root demands protection from the constant action of the oral muscles and the violent assaults that must accompany mastication. When this is not given, if even temporary success be secured it is done despite the forbidding conditions. If permanency is to be attained in such cases, everything that will contribute to a favoring condition should be done, and the immovability of the implanted tooth should first of all be secured. Systemic treatment may be required, and if this be not attended to the continued tenure of the case will be extremely problematical, and it will become a victim to the first unfavoring complication which arises.

A careful study of the excellent paper in this number by Dr. Miller, will throw light upon the question. His experiments, as there detailed, are very instructive, for they are absolute demonstrations and not speculative hypotheses.

